# Early emollient bathing is associated with subsequent atopic dermatitis in an unselected birth cohort study

**DOI:** 10.1111/pai.13998

**Published:** 2023-07-18

**Authors:** Cathal O'Connor, Vicki Livingstone, Jonathan O’B Hourihane, Alan D. Irvine, Geraldine Boylan, Deirdre Murray

**Affiliations:** ^1^ Paediatrics and Child Health Cork University Hospital Cork Ireland; ^2^ INFANT Research Centre University College Cork Cork Ireland; ^3^ Paediatrics and Child Health, Royal College of Physicians of Ireland Dublin Ireland; ^4^ Department of Dermatology Children's Health Ireland at Crumlin Dublin Ireland

**Keywords:** atopic dermatitis, early intervention, emollient, emollient bathing, prevention, skin barrier

## Abstract

**Background:**

Skin barrier dysfunction is a key component of the pathogenesis of atopic dermatitis (AD). Recent research on barrier optimization to prevent AD has shown mixed results. The aim of this study was to assess the relationship between emollient bathing at 2 months and the trajectory of AD in the first 2 years of life in a large unselected observational birth cohort study.

**Methods:**

The Babies After SCOPE: Evaluating the Longitudinal Impact Using Neurological and Nutritional Endpoints Birth Cohort study enrolled 2183 infants. Variables extracted from the database related to early skincare, skin barrier function, parental history of atopy, and AD outcomes. Statistical analysis was performed to adjust for potential confounding variables.

**Results:**

One thousand five hundred five children had data on AD status available at 6, 12, and 24 months. Prevalence of AD was 18.6% at 6 months, 15.2% at 12 months, and 16.5% at 24 months. Adjusted for potential confounding variables, the odds of AD at any point were higher among infants who had emollient baths at 2 months (OR (95% CI): 2.41 (1.56 to 3.72), *p* < .001). Following multivariable analysis, the odds of AD were higher among infants who had both emollient baths and frequent emollient application at 2 months, compared with infants who had neither (OR (95% CI) at 6 months 1.74 (1.18–2.58), *p* = .038), (OR (95% CI) at 12 months 2.59 (1.69–3.94), *p* < .001), (OR (95% CI) at 24 months 1.87 (1.21–2.90), *p* = .009).

**Conclusion:**

Early emollient bathing was associated with greater development of AD by 2 years of age in this population‐based birth cohort study.

**Key Message**

Skin barrier dysfunction is a key pathogenic mechanism in atopic dermatitis (AD). Recent research on emollient use for barrier optimization in the first year of life has shown mixed results in the prevention of AD. Little is known about the impact of emollient bathing in very early life on AD outcomes. In this large observational cohort study, emollient bathing at 2 months of age was associated with subsequent development of AD at a population level. This was consistent after adjusting for family history, transepidermal water loss, and early signs of dermatitis. Future preventative research should focus on specific emollient practices and high‐risk populations and other factors such as immune responses and microbial exposure.

## 1 INTRODUCTION

Atopic dermatitis (AD) affects one in five children^1^ and has a high disease burden.^2^ AD usually starts in the first year of life and disease activity commonly persists into adulthood.^3^ AD is strongly associated with multiple comorbidities,^4^ including atopic diseases, such as food allergy,^5^ asthma,^6^ and allergic rhinitis,^7^ and psychological illness such as anxiety, depression, and attention‐deficit/hyperactivity disorder.^8^, ^9^

The pathophysiology of AD is complex, involving skin barrier dysfunction,^10^ aberrant immune responses,^11^ and environmental factors such as microbial exposure.^12^ Loss‐of‐function mutations in *FLG* (encoding filaggrin, which contributes to skin barrier integrity),^13^ represent the greatest genetic risk factor for AD.^14^ Emollients have been targeted to repair skin barrier defects in AD. Two small pilot trials showed that early emollient application to infants at high risk of developing AD reduced the development of AD significantly.^15^, ^16^ However, two randomized controlled trials (RCT) showed no benefit in early emollient use,^17^, ^18^ although a more recent RCT showed a significant reduction in the development of AD in high‐risk infants if emollients were applied between birth and 2 months and then stopped.^19^ A Cochrane review evaluating skin care interventions concluded that, based on low‐moderate certainty evidence, skin care interventions such as emollients during the first year of life in healthy infants are probably not effective for preventing eczema and probably increase the risk of skin infection.^20^

We aimed to assess the impact of early emollient bathing on AD development over the first 2 years of life in a large observational birth cohort study.

## 2 METHODS

### 2.1 Study subjects

This study was a secondary analysis of the Cork Babies After SCOPE: Evaluating the Longitudinal Impact Using Neurological and Nutritional Endpoints (BASELINE) Birth Cohort study (http://www. baselinestudy.net/). The purpose of BASELINE was to examine the effects of environmental exposures during pregnancy and infancy on childhood health and development. BASELINE recruited healthy first‐born term babies and performed assessments at birth, 2, 6, 12, and 24 months involving parental questionnaires and physical assessment, including serial skin barrier assessment using transepidermal water loss (TEWL) measurements. Questionnaires at 2 months included questions about bathing and moisturizing habits, including frequency of bathing and moisturizing, and products used; and a specific screening question for AD (“Has you baby had an itchy rash on the face or in folds of arms or legs?”). Emollient baths were defined as baths with oil or emulsifier‐based additives. Emollient application was defined as the application of a “leave on” emollient directly onto the skin. Parental atopy was defined as a self‐reported parental history of AD, asthma, or allergic rhinitis. Experienced healthcare professionals diagnosed AD at 6, 12, and 24 months using validated diagnostic criteria.^21^, ^22^, ^23^ TEWL measurements were carried out using a validated open chamber system (Tewameter TM 300; Courage + Khazaka Electronic).

### 2.2 Data extraction

Data extracted from the database included: sex, birthweight, gestational age, and parental atopy; frequency of moisturizing, frequency of bathing, use of emollient bath additives, presence of parent‐reported “itchy rash,” and TEWL at 2 months; and AD status at 6, 12, and 24 months.

### 2.3 Statistical analysis

Categorical variables were described using frequency (percentage) and continuous variables using mean (standard deviation (SD)). To investigate whether changes in AD prevalence were influenced by emollient bathing at 2 months, a mixed effects logistic regression model was used. Time points (6, 12, and 24 months), emollient bathing at 2 months (yes, no) and their interaction (time*emollient use) were categorical fixed effects in the model. If the interaction term was not significant, the model was rerun with the interaction term removed. To adjust for potential confounding variables, the model (without the interaction term) was rerun with the potential confounding variables included as fixed effects.

Univariable and multivariable logistic regression models were used to investigate relationships between use of emollient baths and frequency of emollient application at 2 months and the presence of AD at 6, 12, and 24 months of age separately. To investigate whether the relationship between emollient bathing/application and AD differed by itchy skin status at 2 months, the interaction term emollient use*itchy skin was included in a multivariable model.

Infants were grouped according to the combination of emollient bathing and emollient application practices and univariable and multivariable logistic regression was performed. Models were run with those with “itchy rash” at 2 months included and then excluded. Emollient bathing was divided into those who had emollient baths at 2 months versus those who did not. Emollient application was divided into those who had emollient applied more than once weekly (“frequent”) versus those who had emollient applied once a week or less (“infrequent”). Variables were combined to create four categories:

1. Emollient bath, frequent emollient application.
2. Emollient bath, infrequent emollient application.
3. No emollient bath, frequent emollient application.
4. No emollient bath, infrequent emollient application.

The potential confounders included in all multivariable models were sex, birth weight, bathing frequency at 2 months, TEWL at 2 months, “itchy rash” at 2 months, and presence of maternal/paternal atopy (AD, asthma, rhinitis). For all independent variables, unadjusted and adjusted odds ratios (ORs) and 95% confidence intervals (95% CIs) are presented. Prior to performing the multivariable analyses, multicollinearity among the independent variables was tested using the variance inflation factor. All tests were two‐sided, and a *p*‐value <.05 was considered statistically significant. Statistical analysis was performed using Stata (version 15.1, StataCorp LP).

## 3 RESULTS

BASELINE recruited 2183 infants; 50% were male, and 98% were Caucasian. Mean birthweight was 3489 g (SD = 512 g), and mean gestation was 39.9 weeks (SD = 1.5 weeks).^24^

### 3.1 Changes in atopic dermatitis status over time (6, 12, and 24 months)

Data on AD status at all three time points (6, 12, and 24 months) were available for 1505 children. Prevalence of AD was 18.6% (280/1505) at 6 months, 15.2% (229/1505) at 12 months, and 16.5% (249/1505) at 24 months. Almost 11% of children (10.8%, 163/1505) had persistent AD (AD at six and/or 12 and 24 months), 13.0% (196/1505) had AD at 6 and/or 12 months, but not at 24 months, 5.7% (86/1505) did not have AD at 6 or 12 months but had AD at 24 months, and 70.4% (1060/1505) never had AD.

Of 1505 infants, data on emollient bathing at 2 months of age and potential confounders were available for 1296 infants. At 2 months, 28.2% (365/1296) of infants had emollient baths. Changes in AD prevalence from 6 to 24 months were not influenced by emollient bathing at 2 months (*p* = .570). After removal of the interaction term, both time point (*p* = .010) and emollient bathing (*p* < .001) were statistically significant. The odds of AD were higher among infants who had emollient baths at 2 months (OR (95% CI): 3.18 (2.04–4.94)). After adjustment for potential confounders, both time point (*p* = .011) and emollient bathing (*p* < .001) remained statistically significant, with the odds of AD at any time point higher among infants who had emollient baths at 2 months (OR (95% CI): 2.41 (1.56–3.72)).

### 3.2 Effect of emollient bathing at 2 months on atopic dermatitis at 6 months

**FIGURE 1:**
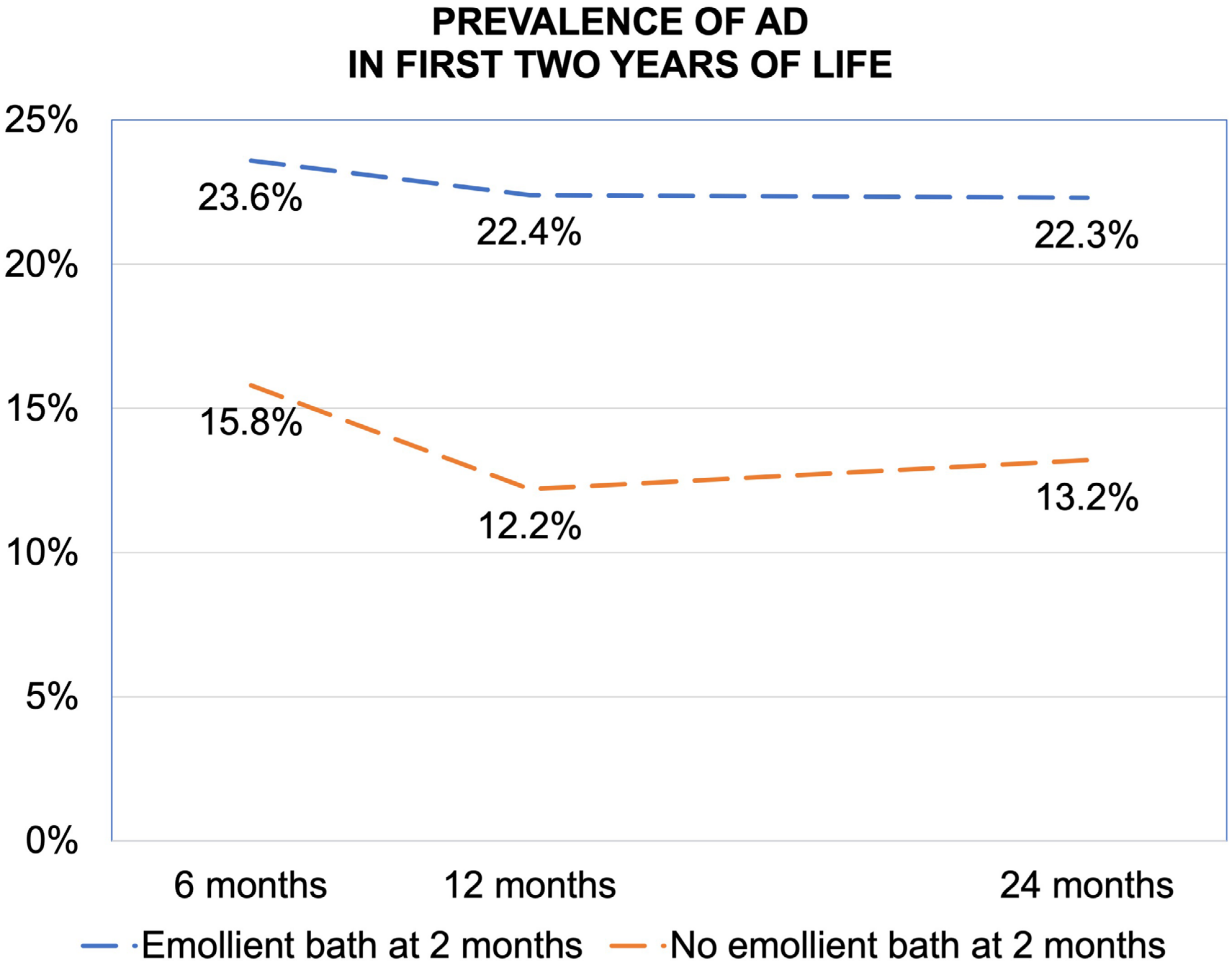
Prevalence of atopic dermatitis (AD) over the first 2 years of life according to the use of emollient baths at 2 months of age. At 6 months *n* = 1538, at 12 months *n* = 1452, and at 24 months *n* = 1332.

At 6 months, 1538 infants were included in the analysis. The prevalence of AD in those who had emollient baths at 2 months was 23.6% (101/428), compared with 15.8% (175/1110) in those who did not have emollient baths (Figure 1).

In the univariable analysis (Table S1), daily emollient application at 2 months (OR (95% CI): 1.72 (1.25–2.36)), and emollient bathing at 2 months (OR (95% CI): 1.65 (1.25–2.17)) were significantly associated with AD at 6 months. In the multivariable analysis (Table 1), emollient bathing at 2 months remained significantly associated with AD at 6 months (adjusted OR (95% CI): 1.41 (1.05–1.89)). The relationship between early emollient bathing and AD at 6 months was independent of whether the infant had itchy skin at 2 months (*p* = .092).

**TABLE 1:** Results of the multivariable logistic regression analyses with atopic dermatitis (AD) at 6 months (*n* = 1538), 12 months (*n* = 1452), and 24 months (*n* = 1332), as the dependent variables.

	AD at 6 months		Multivariable		AD at 12 months		Multivariable		AD at 24 months		Multivariable	
	Yes (*n* = 276)	No (*n* = 1262)	OR	*p*‐value	Yes (*n* = 219)	No (*n* = 1233)	OR	*p*‐value	Yes (*n* = 210)	No (*n* = 1122)	OR	*p*‐value
	*n* (%)	*n* (%)	(95% CI)		*n* (%)	*n* (%)	(95% CI)		*n* (%)	*n* (%)	(95% CI)	
Frequency of emollient application at 2 months												
Once weekly or less	116 (42.0)	619 (49.1)	1	.225	86 (39.3)	614 (49.8)	1	**.039**	94 (44.8)	562 (50.1)	1	.917
2–3 times weekly	78 (28.3)	388 (30.7)	1.07 (0.77–1.50)		62 (28.3)	369 (29.9)	1.16 (0.80–1.68)		60 (28.6)	327 (29.1)	1.07 (0.74–1.56)	
Daily or several times daily	82 (29.7)	255 (20.2)	1.36 (0.96–1.93)		71 (32.4)	250 (20.3)	1.64 (1.12–2.40)		56 (26.7)	233 (20.8)	1.07 (0.72–1.61)	
Frequency of bathing at 2 months												
Once weekly or less	90 (32.6)	390 (30.9)	1	.906	71 (32.4)	392 (31.8)	1	0.620	69 (32.9)	359 (32.0)	1	.969
2–3 times weekly	125 (45.3)	616 (48.8)	0.94 (0.68–1.30)		103 (47.0)	592 (48.0)	1.01 (0.71–1.44)		94 (44.8)	541 (48.2)	0.99 (0.69–1.43)	
More than 3 times weekly	61 (22.1)	256 (20.3)	0.92 (0.62–1.37)		45 (20.6)	249 (20.2)	0.83 (0.53–1.30)		47 (22.4)	222 (19.8)	1.04 (0.67–1.62)	
Emollient bath additive used at 2 months												
No	175 (63.4)	935 (74.1)	1	**.023**	127 (58.0)	915 (74.2)	1	**<.001**	126 (60.0)	830 (74.0)	1	**.001**
Yes	101 (36.6)	327 (25.9)	1.41 (1.05–1.89)		92 (42.0)	318 (25.8)	1.84 (1.34–2.52)		84 (40.0)	292 (26.0)	1.70 (1.23–2.35)	

*Note*: Multivariable analysis includes the variables listed and sex, birth weight, TEWL at 2 months, “itchy rash” at 2 months and presence of maternal/paternal atopy (AD, asthma, or rhinitis).

Values in bold represent statistically significant results (*p* < .05).

### 3.3 Effect of emollient bathing at 2 months on AD at 12 months

At 12 months, 1452 infants were included in the analysis. The prevalence of AD in those who had emollient baths at 2 months was 22.4% (92/410), compared with 12.2% (127/1042) in those who did not have emollient baths (Figure 1).

In the univariable analysis (Table S1), daily emollient application at 2 months (OR (95% CI): 2.03 (1.43–2.87)) and emollient bathing at 2 months (OR (95% CI): 2.08 (1.55–2.81)) were significantly associated with AD at 12 months. In the multivariable analysis (Table 1), daily or multiple daily emollient application at 2 months (adjusted OR (95% CI): 1.64 (1.12–2.40)) and emollient bathing at 2 months (adjusted OR (95% CI): 1.84 (1.34–2.52)) remained significantly associated with AD at 12 months. The relationship between early emollient bathing and AD at 12 months was independent of whether the infant had itchy skin at 2 months (*p* = .362).

### 3.4 Effect of emollient bathing at 2 months on AD at 24 months

At 24 months, 1332 children were included in the analysis. The prevalence of AD in those who had emollient baths at 2 months was 22.3% (84/376), compared with 13.2% (126/956) in those who did not have emollient baths (Figure 1).

In the univariable analysis (Table S1), emollient bathing at 2 months (OR (95% CI): 1.89 (1.39–2.57)) was significantly associated with AD at 24 months. In the multivariable analysis (Table 1), emollient bathing at 2 months remained significantly associated with AD at 24 months (adjusted OR (95% CI): 1.70 (1.23–2.35)). The relationship between early emollient bathing and AD at 24 months did not depend on whether the child had itchy skin at 2 months (*p* = .865).

### 3.5 Outcomes of combination emollient bathing and application at 2 months

**FIGURE 2:**
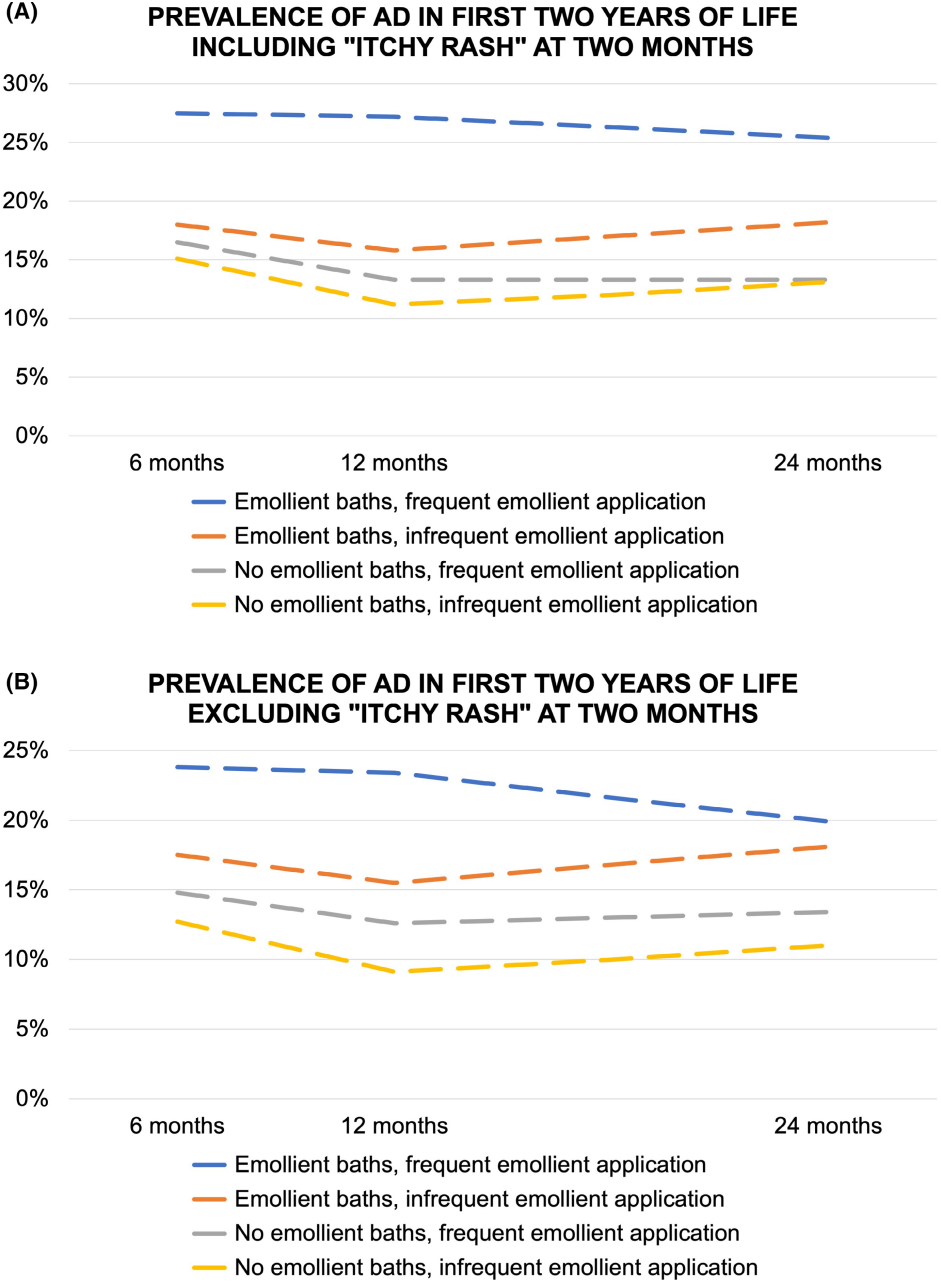
(A) Prevalence of atopic dermatitis (AD) in the first 2 years of life stratified according to the use of emollient baths and frequency of emollient application, including infants with “itchy rash” at 2 months. At 6 months *n* = 1538, at 12 months *n* = 1452, and at 24 months *n* = 1332. (B) Prevalence of AD in the first 2 years of life stratified according to the use of emollient baths and frequency of emollient application, excluding infants with “itchy rash” at 2 months. At 6 months *n* = 1395, at 12 months *n* = 1315, and at 24 months *n* = 1201.

Subjects were further grouped according to the combination of emollient bathing and application practices. At all time points, the prevalence of AD was highest in the group who had emollient baths and frequent emollient application, with similar trends seen if infants with “itchy rash” at 2 months were excluded (Table 2, Figure 2A,B).

**TABLE 2:** Prevalence of atopic dermatitis (AD) at 6, 12, and 24 months with combinations of use of emollient baths (yes/no) and frequency of emollient application (frequent defined as more than once weekly, infrequent defined as once weekly or less) at 2 months, including and excluding infants with “itchy rash” at 2 months.

AD including infants with “itchy rash” at 2 months						
	At 6 months		At 12 months		At 24 months	
	Yes (*n* = 276)	No (*n* = 1262)	Yes (*n* = 219)	No (*n* = 1233)	Yes (*n* = 210)	No (*n* = 1122)
	*n* (%)	*n* (%)	*n* (%)	*n* (%)	*n* (%)	*n* (%)
Group 1	69 (25.0)	182 (14.4)	65 (29.7)	174 (14.1)	55 (26.2)	162 (14.4)
Group 2	32 (11.6)	145 (11.5)	27 (12.3)	144 (11.7)	29 (13.8)	130 (11.6)
Group 3	91 (33.0)	461 (36.5)	68 (31.1)	445 (36.1)	61 (29.1)	398 (35.5)
Group 4	84 (30.4)	474 (37.6)	59 (26.9)	470 (38.1)	65 (31.0)	432 (38.5)

AD excluding infants with “itchy rash” at 2 months						
	At 6 months		At 12 months		At 24 months	
	Yes (*n* = 218)	No (*n* = 1177)	Yes (*n* = 174)	No (*n* = 1141)	Yes (*n* = 168)	No (*n* = 1033)
	*n* (%)	*n* (%)	*n* (%)	*n* (%)	*n* (%)	*n* (%)
Group 1	48 (22.0)	154 (13.1)	45 (25.9)	147 (12.9)	34 (20.2)	137 (13.3)
Group 2	29 (13.3)	137 (11.6)	25 (14.4)	136 (11.9)	27 (16.1)	122 (11.8)
Group 3	75 (34.4)	431 (36.6)	59 (33.9)	408 (35.8)	56 (33.3)	361 (35.0)
Group 4	66 (30.3)	455 (38.7)	45 (25.9)	450 (39.4)	51 (30.4)	413 (40.0)

*Note*: Group 1 = emollient bath, frequent emollient application; Group 2 = emollient bath, infrequent emollient application; Group 3 = no emollient bath, frequent emollient application; and Group 4 = no emollient bath, infrequent emollient application.

Univariable and multivariable logistic regression analysis showed similar findings, with significantly higher odds ratios in infants who had emollient baths and frequent emollient application, compared with infants who had no emollient baths and infrequent emollient application (Table 3). The findings were similar when infants with “itchy rash” at 2 months were excluded.

**TABLE 3:** Odds ratios (OR) and 95% confidence intervals (95% CI) for atopic dermatitis (AD) at 6, 12, and 24 months for infants who had emollient baths and frequent emollient application, relative to infants who had no emollient baths and infrequent emollient application (frequent defined as more than once weekly, infrequent defined as once weekly or less) at 2 months, including and excluding infants with “itchy rash” at 2 months.

		6 months OR (95% CI)	*p*‐value	12 months OR (95% CI)	*p*‐value	24 months OR (95% CI)	*p*‐value
**AD including infants with “itchy rash” at 2 months**							
Emollient baths, frequent emollient application	Univariable OR (95% CI)	2.14 (1.49–3.07)	<.001	2.98 (2.01–4.41)	<.001	2.26 (1.51–3.37)	<.001
	Multivariable^a^ OR (95% CI)	1.74 (1.18–2.58)	.038	2.59 (1.69–3.94)	<.001	1.87 (1.21–2.90)	.009
**AD excluding infants with “itchy rash” at 2 months**							
Emollient baths, frequent emollient application	Univariable OR (95% CI)	2.15 (1.42–3.25)	.003	3.06 (1.95–4.82)	<.001	2.01 (1.25–3.23)	.015
	Multivariable^a^ OR (95% CI)	2.14 (1.39–3.30)	.006	3.07 (1.91–4.92)	<.001	2.08 (1.27–3.42)	.021

^a^ Includes the variables listed and sex, birth weight, TEWL at 2 months, “itchy rash” at 2 months, frequency of bathing at 2 months, and presence of maternal/paternal atopy (AD, asthma, or rhinitis).

### 3.6 Impact of emollient practices at 2 months on TEWL

A linear mixed model was used to investigate whether changes in TEWL between 2 days and 2 months differed by emollient bathing/application. The fixed effects included in the model were group, time, and the interaction of group by time.

At 2 days, the median (IQR) TEWL was 7.00 g/m^2^/h (5.00–9.00) in the group that did not go on to have emollient baths (*n* = 1097) and 7.23 g/m^2^/h (5.00–9.25) in the group that did go on to have emollient baths (*n* = 422). At 2 months, the median (IQR) was 9.32 g/m^2^/h (7.00–12.01) in the group that did not have emollient baths (*n* = 1215) and 9.66 g/m^2^/h (7.15–13.26) in the group that did use emollients (*n* = 457). In the linear mixed model, the interaction between group and time was not statistically significant (*p* = .797), indicating that the changes in TEWL over time did not differ by emollient bathing practice.

The groups were further divided according to frequency of emollient application (once weekly or less vs. greater than once weekly) and emollient bathing practice (yes vs. no; Table 4). TEWL was significantly higher in the group that had emollient baths and had emollient application greater than once weekly compared to the other three groups (*p* = .003 for group in linear mixed model excluding the interaction group*time).

**TABLE 4:** Summary of the transepidermal water loss values for each group at 2 days and 2 months. Infrequent moisturizing is defined as the use of emollient application once weekly or less.

	2 days		2 months	
	*n*	Median (IQR)	*n*	Median (IQR)
Infrequent moisturizer, no emollient baths	531	7.00 (5.00–9.00)	594	8.99 (6.95–11.80)
Frequent moisturizer, no emollient baths	565	7.00 (5.20–9.00)	621	9.56 (7.26–12.17)
Infrequent moisturizer, emollient baths	166	7.04 (5.04–9.37)	187	8.52 (6.67–12.23)
Frequent moisturizer, emollient baths	256	7.71 (5.00–9.16)	270	10.05 (7.50–13.99)

*Note*: Frequent moisturizing is defined as the use of emollient application greater than once weekly.

### 3.7 Effect of parental history of atopy on outcomes

Atopic dermatitis outcomes were also examined in relation to the use of emollient baths in those with a parental history of atopy (AD/asthma/rhinitis) versus those without a parental history of atopy. At 6 months, the relationship between emollient bathing and increased risk of AD did not differ by history of parental atopy (*p* = .685 for the interaction term). At 12 months, the relationship between emollient bathing and increased risk of AD did differ by history of parental atopy (*p* = .013 for the interaction term). The odds ratio for AD with emollient bathing at 12 months was 2.86 (95% CI: 1.94–4.21) with a parental history of AD and 1.28 (95% CI: 0.78–2.10) without a parental history of AD. At 24 months, the relationship between emollient bathing and increased risk of AD did not differ by history of parental atopy (*p* = .854 for the interaction term).

## 4 DISCUSSION

This secondary analysis of a large unselected first‐born birth cohort showed that emollient bathing at 2 months was associated with the development of AD by 2 years of age. Results were consistent even after accounting for confounding factors such as TEWL at 2 months, parent‐reported “itchy rash” at 2 months, and parental atopy. The relationship was similar at 6, 12, and 24 months, with similar trajectories for infants with an early “itchy rash” versus those without. Over a quarter of infants had emollient baths at 2 months, without being advised to do so by healthcare professionals. Bathing frequency was not associated with the development of AD.

Strengths of this study include the large sample size, unselected nature of the subjects, detailed assessments, and extended follow‐up. Retention between 6 months (*n* = 1538) and 24 months (*n* = 1332) was high at 86.6%. TEWL was calculated using a detailed protocol, and AD was assessed by trained researchers using validated diagnostic criteria. Follow‐up to 24 months excluded transient eczematous eruptions that are common in the first year of life, and most patients who develop AD do so by 24 months. Confounding factors were controlled for as far as possible. Delivery by cesarean section was not shown to be associated with AD in this cohort.^25^

Limitations include the lack of randomization, so early emollient use may have been a parental response to signals of impending AD. However, the statistical model controlled for the presence of an “itchy rash” at 2 months and upper quartile TEWL at 2 months, a functional marker of skin barrier integrity. In addition, subgroup exclusion of infants reported as having an “itchy rash” at 2 months may have helped to exclude infants who had emollient baths due to parental concerns about dry skin and impending AD. Early application of emollients may have masked subclinical AD at 2 months, which became apparent at subsequent time points due to progression in AD activity. The rate of dropout from the study may have differed between the groups who used emollients versus those who did not and between the groups who developed AD and those who did not. The duration of use of emollient baths or emollient application was also not captured, and effects may have been greater with longer use.

The type of emollient/moisturizer was not specified, and products may vary in their effects. The most frequently used emollient on young infants at the time of this cohort study was vegetable oil (30%),^26^ and olive oil is known to impede the lamellar structure of the epidermal barrier.^27^ The composition of the emollient/moisturizer may also be important as the local water quality in Cork is classified as hard to very hard.^28^ Surfactants such as sodium laureth sulfate deposit more easily on the skin when used with hard water, with an associated increase in TEWL and potential for irritation in individuals with normal skin, and more severe symptoms in patients with AD associated with filaggrin mutations.^29^ Thus, the findings may not be as relevant for regions were bathing water is less hard. Parental history of AD, asthma, and rhinitis was self‐reported and therefore may not be completely reliable. This was a monocentric study with a relatively homogenous population.

The findings support recent RCT that show that emollients as bath additives or “leave on” products did not prevent the development of AD.^17^, ^18^ The PreventADALL trial showed an increased risk of AD by 12 months in infants randomized to regular emollient bathing between 2 weeks and 8 months, with an odds ratio of 1.57 (95% CI: 1.10, 2.23).^18^, ^20^ Emollient bathing may interfere with natural lipids in the stratum corneum that reduce TEWL and protect against pathogens, especially in areas where water is classified as hard or very hard. While previous studies have shown that emollient baths have minimal benefit in the treatment of established AD,^30^ this study suggests that early emollient bathing is also associated with higher rates of subsequent AD. However, it cannot be concluded from this study that emollient bathing increases the risk of AD, as the higher rate of AD in emollient users can be explained by several factors. This study showed a similarly increased risk of AD with emollient bathing with or without a parental history of AD. A more controlled study is required to determine the relative effects of different exposures in early life.

It is unclear why previous trials of early emollient application showed a protective effect against AD. The emollient may have masked signs of the developing dermatitis, and the duration of follow‐up may have been insufficient to capture those cases that emerged once emollient therapy was ceased. Another hypothesis is that certain emollients may provide benefit, and the petroleum‐based emollients used in these trials were not protective. Early emollient application may benefit infants at higher risk of AD, such as those with family history or filaggrin mutations, but not benefit infants without skin barrier dysregulation. A recent RCT in our center showed that early initiation of daily specialized emollient application until 2 months reduced the incidence of AD in the first year of life in high‐risk infants.^19^ There may be a key temporal window when early use of emollients is beneficial, and future studies should examine this possibility. This study only assessed the use of emollients prior to the development of AD, and the results do not undermine the efficacy of emollient application for relief of itch and inflammation in established AD.

Future research should focus on specific emollient bathing at specific time points in specific populations, and other preventative targets related to the pathophysiology of AD, such as the microbiome and inflammatory immune pathways.

## AUTHOR CONTRIBUTIONS

**Cathal O'Connor:** Conceptualization; investigation; writing – original draft; methodology; validation; visualization; writing – review and editing; software; formal analysis; project administration; data curation; supervision; resources. **Vicki Livingstone:** Writing – original draft; writing – review and editing; formal analysis; investigation; methodology; validation; software; data curation. **Jonathan O'B Hourihane:** Writing – original draft; writing – review and editing; funding acquisition; investigation; methodology; supervision; resources; project administration. **Alan Irvine D:** Investigation; funding acquisition; writing – original draft; methodology; writing – review and editing; project administration; supervision; resources. **Geraldine Boylan:** Investigation; funding acquisition; writing – original draft; methodology; writing – review and editing; project administration; supervision; resources. **Deirdre Murray:** Investigation; funding acquisition; writing – original draft; methodology; writing – review and editing; resources; supervision; project administration.

## FUNDING INFORMATION

This publication has emanated from research supported in part by a research grant from Science Foundation Ireland (SFI) under Grant Number 12/RC/2272 and 15/SP/3091 and Johnson & Johnson Santé Beauté France, which had no direct involvement in the research. The Cork BASELINE Birth Cohort Study (ClinicalTrials.gov NCT01498965) is supported by the National Children's Research Centre, Dublin, Ireland, and by the Food Standards Agency, United Kingdom (TO7060). COC is funded by the Irish Clinical Academic Training (ICAT) program, supported by the Wellcome Trust and the Health Research Board (grant number 223047/Z/21/Z); the Health Service Executive National Doctors Training and Planning; and the Health and Social Care, Research and Development Division, Northern Ireland.

## CONFLICT OF INTEREST STATEMENT

JO'BH receives research funding related to this field from the City of Dublin Skin and Cancer Hospital Charity and to unrelated research projects from Clemens von Pirquet Foundation and Temple St Hospital Research Foundation and Johnson & Johnson, research funding and speaker fees and consultancy fees from Aimmune Therapeutics, research funding and speaker fees from DBV Technologies.

5

### PEER REVIEW

The peer review history for this article is available at https://www.webofscience.com/api/gateway/wos/peer‐review/10.1111/pai.13998.

## Supporting information


Table S1
