# Supplementary material for: Early emollient bathing is associated with subsequent atopic dermatitis in an unselected birth cohort study
Source: Pediatr Allergy Immunol. 2023 Jul 18;34(7):e13998. doi: 10.1111/pai.13998 (PMC10947084; doi:10.1111/pai.13998)
Supplement: Supplementary file 1 — Table S1 [file PAI-34-0-s001.docx]

**Tables**

|  | AD at 6 months | | Univariable | | AD at 12 months | | Univariable | | AD at 24 months | | Univariable | |
| --- | --- | --- | --- | --- | --- | --- | --- | --- | --- | --- | --- | --- |
|  | Yes (n=276) | No (n=1262) | OR | p-value | Yes (n=219) | No (n=1233) | OR | p-value | Yes (n=210) | No (n=1122) | OR | p-value |
|  | n (%) | n (%) | (95%CI) |  | n (%) | n (%) | (95%CI) |  | n (%) | n (%) | (95%CI) |  |
| Frequency of emollient application at 2 months |  | |  | 0.003 |  |  |  | <0.001 |  |  |  | 0.146 |
| Once weekly or less | 116 (42.0) | 619  (49.1) | 1 |  | 86  (39.3) | 614  (49.8) | 1 |  | 94  (44.8) | 562  (50.1) | 1 |  |
| 2-3 times weekly | 78  (28.3) | 388  (30.7) | 1.07  (0.78-1.47) |  | 62  (28.3) | 369  (29.9) | 1.20  (0.84-1.70) |  | 60  (28.6) | 327  (29.1) | 1.10  (0.77-1.56) |  |
| Daily or several times daily | 82  (29.7) | 255  (20.2) | 1.72  (1.25-2.36) |  | 71  (32.4) | 250  (20.3) | 2.03  (1.43-2.87) |  | 56  (26.7) | 233  (20.8) | 1.44  (1.00-2.07) |  |
| Frequency of bathing at 2 months |  |  |  | 0.561 |  |  |  | 0.965 |  |  |  | 0.585 |
| Once weekly or less | 90  (32.6) | 390  (30.9) | 1 |  | 71  (32.4) | 392  (31.8) | 1 |  | 69  (32.9) | 359  (32.0) | 1 |  |
| 2-3 times weekly | 125 (45.3) | 616  (48.8) | 0.88  (0.65-1.19) |  | 103 (47.0) | 592  (48.0) | 0.96  (0.69-1.33) |  | 94  (44.8) | 541  (48.2) | 0.90  (0.64-1.27) |  |
| More than 3 times weekly | 61  (22.1) | 256  (20.3) | 1.03  (0.72-1.48) |  | 45  (20.6) | 249  (20.2) | 1.00  (0.66-1.50) |  | 47  (22.4) | 222  (19.8) | 1.10  (0.73-1.65) |  |
| Emollient bath additive used at 2 months |  |  |  | <0.001 |  |  |  | <0.001 |  |  |  | <0.001 |
| No | 175 (63.4) | 935  (74.1) | 1 |  | 127 (58.0) | 915  (74.2) | 1 |  | 126 (60.0) | 830  (74.0) | 1 |  |
| Yes | 101 (36.6) | 327  (25.9) | 1.65  (1.25-2.17) |  | 92  (42.0) | 318  (25.8) | 2.08  (1.55-2.81) |  | 84  (40.0) | 292  (26.0) | 1.89  (1.39-2.57) |  |

Supplementary Table 1. Results of the univariable logistic regression analyses with AD at six months (n=1538), 12 months (n=1452), and 24 months (n=1332), as the dependent variable.
